# Effect of Steam Blanching, Dehydration Temperature & Time, on the Sensory and Nutritional Properties of a Herbal Tea Developed from *Moringa oleifera* Leaves

**DOI:** 10.1155/2020/5376280

**Published:** 2020-01-10

**Authors:** Yasara W. H. Wickramasinghe, Indira Wickramasinghe, Isuru Wijesekara

**Affiliations:** Department of Food Science & Technology, Faculty of Applied Sciences, University of Sri Jayewardenepura, Nugegoda, Sri Lanka

## Abstract

The core purpose of the current study is to explore the use of *Moringa oleifera* leaves, to produce a herbal tea with acceptable sensory properties and nutritional properties by utilizing the steam blanching technique, different dehydration temperatures and time, which can be accepted in the Sri Lankan market. Six sets of samples were prepared where temperature and time combinations were 55°C—6 h, 60°C—4.30 h, 65°C—3 h for the unblanched samples & 55°C—6 h, 60°C—5.30 h & 65°C—4 h for the steam blanched samples. These samples were evaluated, employing a trained panel of 5 tea tasters and a semi trained panel of 35 members. The sample code 706 (steam blanched, 65°C—4 h) was selected as the sample with best sensory attributes. The blanched and unblanched samples dried at 65°C were tested for their proximate, mineral, vitamin, antioxidant and phytochemical contents. The effects of steam blanching on these two samples were evaluated & compared. This study highlights that steam blanching significantly increased the carbohydrates, fat, Mn, Fe, vitamin A, vitamin E and the DPPH scavenging activity whereas steam blanching significantly reduced the protein, fiber, Na, K, Ca, Total phenolic contents and flavonoids content but vitamin C, Zn, Cu and Mg contents were unaffected by steam blanching.

## 1. Introduction

The drinking of tea began in China centuries ago, and has over the years become an inseparable part of most cultures worldwide. Tea is currently one of the most widely consumed beverages in the world and therefore ranks as an important food product. Tea holds a unique place in the culture of many societies. Tea is an infusion of dried leaves and buds of the plant *Camellia sinensis* and it is predominantly consumed due to its attractive aroma and taste as well its immense health benefits [1]. Tea has been traditionally categorized into black tea, green tea, oolong tea, and earl gray tea according to different processing methods [2].

In the recent years, a fourth category called the “herb tea” has been growing popularity among consumers. Herb tea or herbal tea is defined as an infusion made from herbs, fruits, flowers, stems, roots, etc. made from plant parts other than *Camellia sinensis *[2]. Such teas are usually caffeine-free tea.

The use of Cinnamon leaves & cloves, Citronella leaves, Roselle calices, Peppermint, Chamomile, Ginger, Lemon, Jasmine, Milk thistle, Rosehip, Hibiscus, Dandelion, Fennel and other indigenous herbs in making herbal tea has become a common practice [3, 4]. Currently, there is growing interest in the use of *Moringa* leaves as an ingredient in the preparation of herbal tea due to its health benefits. Herbal teas are growing popularity due to the fact that public is showing much interest in the use of herbal remedies [5].

*Moringa* is truly a miracle plant, for the nourishing and healing of man. In the recent years it has come to be known as a “multipurpose” plant [6]. *Moringa* is the sole genus in the flowering plant family Moringaceae. The genus *Moringa* is made up of 13 species. The species most common, and which is the main subject of this study is the species called “*Moringa oleifera*.” *Moringa oleifera* is found in many tropical and sub-tropical regions. All parts of the *Moringa* tree are edible and have long been consumed by humans [7].

Every part of *Moringa *is used for certain nutritional and/or medicinal purposes. *Moringa* is a highly nutritious plant which could be used as a good source of nutrition for the people of all age groups [8]. *Moringa* has been found to contain many essential nutrients, such as vitamins, minerals, amino acids, beta-carotene, antioxidants and omega 3 and 6 fatty acids [9]. According to Fahey, this tree has been advocated as an outstanding source of highly digestible protein, Ca, vitamin C and carotenoids which is suitable to be utilized in regions of the world where undernourishment is a major concern [7]. It has been found that *Moringa *leaf tea infusions possess higher amount of minerals than other green/herbal tea infusions and thus it is evident that drinking of *Moringa* tea enhances the dietary intake of minerals [8].

Various aqueous and alcoholic extracts of *Moringa* leaves have been found to show significant cholesterol lowering action, antiulcerogenic effects, hepatoprotective effects, antitumor activity, anticancer activity, anti-inflammatory effects, antioxidant activity, antimicrobial properties, and antidiabetic activities [7, 9–12].

Even though *Moringa* tea is being consumed in other countries, a product development has not been done in Sri Lanka for *Moringa* tea to be used in the local market. *Moringa* plant which is indigenous to Sri Lanka, grows mostly in the dry zones such as Jaffna, Kalpitiya, Mannar, Puttalam and Hambantota, is considered as an underutilized plant in Sri Lanka [12]. Hence developing new herbal tea products from indigenous plants will bring to light the potential of the underutilized plants for food product development. It will further provide consumers with new alternatives to traditional teas.

The sensory appeal of tea, like all foods products, is an important consideration in new product development. Herbal tea in particular, is gaining increasing consumer attention due to a growing awareness of health benefits derived from their consumption. But consumers are generally unwilling to buy food which is poor in sensory appeal, irrespective of health or nutritional benefits [13]. For this reason, a closer attention has to be given to the sensory properties of functional foods in new product development.

Drying is the oldest and the most commonly used technique used for the preservation of vegetables. Drying extends the shelf life of vegetables, by reducing the moisture content to a level where microorganisms cannot grow [14, 15].

Studies have shown temperatures in the range of 50–80°C, to show acceptable sensory attributes and nutritional properties [14, 16]. The study done by Clemet et al., has found 60°C to be the best drying temperature in terms of nutritional content [17]. Thus the drying temperatures of 55°C, 60°C and 65°C were selected for the current study. Drying under these temperatures were carried out for steam blanched and un-blanched leaves. Steam blanching was used as a preliminary step prior to drying, to minimize drying losses and to ensure a complete inactivation of enzymes responsible for oxidation [16, 18]. The purpose of inactivating enzymes is to modify the texture, preserve colour, flavour, nutritional value and to remove trapped air [15].

The core purpose of the current research is to explore the use of *Moringa oleifera* leaves, to produce a herbal tea with acceptable sensory properties and nutritional properties by using the steam blanching technique, different dehydration temperatures and time.

## 2. Materials and Methods

### 2.1. Plant Material

Fresh *Moringa oleifera* leaves were harvested along with the petioles, from Kaluthara district, Horana area in Sri Lanka, in May 2017. Leaves were harvested early in the morning. All wilting and visibly deceased plant materials were removed and the fresh leaves were washed well with running water. The leaves including the stalks were separated from the petioles and were taken to the laboratory of the University of Sri Jayewardenepura, Sri Lanka.

### 2.2. Sample Preparation

#### 2.2.1. Preparation of Herbal Tea Leaves from Unblanched Leaves

Initially the weight of the leaves was obtained and was spread on aluminium trays. The leaves were oven dried at 55°C until the moisture content was less than 7%. Dried leaves were ground in a grinder (Model: Panasonic MX-GM 1011) and the final weight of the leaves was obtained. The ground leaves were then sieved using a mesh of 355 micron. Leaves remaining on the mesh were collected and was stored in air tight glass bottles, in a deep freezer (−3°C) for sensory analysis and chemical analysis. This procedure was repeated at 60°C and 65°C and thus obtained the samples **521, 592 & 563**, respectively.

#### 2.2.2. Preparation of Herbal Tea Leaves from Blanched Leaves

Initially the weight of the leaves was obtained. Then the leaves were steam blanched for 3 minutes, and was cooled using cold water to prevent the overcooking of leaves. Blanched leaves were spread on clean cotton clothes to remove water and the leaves were spread on aluminium trays and was dried at 55°C until the moisture content was less than 7%. Then the dried leaves were ground in a clean grinder (Model: Panasonic MX-GM 1011) and the final weight of the leaves was obtained. The ground leaves were sieved using a mesh of 355 micron and the leaves remaining on the mesh were collected and was stored in air tight glass bottles, in a deep freezer (−3°C) for sensory analysis and chemical analysis as before. This procedure was repeated at 60°C and 65°C and thus obtained the samples **784, 725 & 706**.

### 2.3. pH Measurement

Two grams each, of the six samples, were soaked in 100 ml of boiling water for 30 minutes. Then each sample was drained using a separate cotton cloth strainer. The extract was used to determine the pH directly with the help of a digital pH meter (Model: Consort C6010).

### 2.4. Colour Measurement

The spectrocolorimeter (Model: Lovibond LC 100) was used to measure the L^∗^, a^∗^ and b^∗^ values of the six *Moringa* tea leaf samples and their powders. Before taking the readings, the spectrocolorimeter was standardized using black and white standard plates. Fifteen readings of each sample were taken randomly and the average value was obtained and was represented as mean ± standard deviation.

### 2.5. Sensory Evaluation

Tea samples were prepared using 2 g of dry tea leaves in a muslin tea strainer and adding 150 mL of boiling (100°C) water to it. It was let to brew for five minutes. A trained panel of 5 tea tasters from the southern group of companies, Sri Lanka and a semi trained panel of 35 members from the University of Sri Jayewardenepura, Department of Food Science & Technology, were chosen for the sensory evaluation. The attributes evaluated were dry leaf texture, brew colour, brew aroma, brew taste, astringency, after taste and overall acceptability. A five point hedonic scale was used where 1 represented “dislike very much” and 5 represented “like very much”.

The sensory evaluation took place in the mid-morning (10:30 am–12:30 pm) to ensure that the panelists were neither hungry nor full [19]. Each panelist was provided with a bottle of water and cream cracker biscuits for consumption between intervals of evaluation. The panelists were asked to rinse their mouths before and after each tasting. The panelists were served with 40–50 ml of the each sample in white colour plastic cups, which were coded with three digit random codes [13, 20].

### 2.6. Determination of Nutritional Composition

Proximate, mineral, vitamin, antioxidant and phytochemical compositions were carried out for two samples: the sample that was selected from the sensory analysis and another sample which is dried at the same temperature as the sample which is selected from the sensory analysis. The analyses have been done in triplicates.

#### 2.6.1. Proximate Analysis

Moisture content, ash content, crude fat content and crude fiber content was determined using the standard method of AOAC, 2005 [21]. Total Nitrogen content was determined using the micro-kjeldahl method as described by the standard method of AOAC, 2005 [21]. To obtain the crude protein content, the total nitrogen content was multiplied by the conversion factor of 6.25 [19, 21]. Total carbohydrate content was determined by difference as follows: 100 – (ash % + Protein % + fat % + fiber % + moisture %) [19]. The energy value was calculated using the Atwater factors where protein, fat and carbohydrate supplied were 4, 9, 4 kcal/g respectively [22]. Proximate analysis was carried out on dry weight basis.

#### 2.6.2. Mineral Analysis

The samples (5.0 g) were weighed into clean porcelain crucibles and were subjected to dry ashing in a muffle furnace (model: SHIMADEN SR1) at 550°C for 5 hours. The resultant ash was dissolved in 5.0 ml of HNO_3_/HCl (1:2) and was heated gently on a heating mental until the brown fumes disappeared. Then they were transferred into conical flasks and 5.0 ml of deionized water was added into each flask and was heated until a colourless solution was obtained. The mineral solution was filtered into a 25.0 ml volumetric flask through double filter papers, and was made up to the mark with deionized water. The solutions were analyzed in triplicate for the minerals Na, K, Ca, Mg, Mn, Zn, Fe and Cu using the polarized Zeeman atomic absorption spectrophotometer of the model ZA3000, HITACHI [23].

#### 2.6.3. Vitamin C Analysis

Ascorbic acid content was determined using the 2,6-dichloroindophenol titrimetric method as described by the standard method of AOAC, 2005 [21]. The vitamin content was calculated using the formula below.

(1) $$\begin{array}{c} \text{Vitamin C in mg}/100\text{ml}=\frac{\text{Titre value}\times 4.2\times 100}{\text{Volume of sample}}, \end{array}$$

where, 4.2 = mg ascorbic acid equivalents to 1.0 ml indophenol standard solution.

#### 2.6.4. Vitamin A Analysis

Vitamin A content was determined using the procedure described by [17]. 5.0 g of sample was soaked in 20 ml of acetone overnight and was filtered in to a separating funnel and 10 ml of PET ether was added into it. The resultant solution was shook vigorously and was allowed to stand for some time with the lid open. The top layer was collected and the absorbance of it was taken at 452 nm using the UV–Vis spectrophotometer (model: UV mini-1240 SHIMADZU, serial no: A109347). The absorbance value was inserted into the below formula.

(2) $$\begin{array}{c} \text{Vitamin A}=\frac{3.85\times A\times V\times 100}{W\times 100}, \end{array}$$

where, *A* = absorbance, *V* = volume of PET ether, *W* = weight of the sample.

#### 2.6.5. Vitamin E Analysis

Vitamin E content was determined using the HPLC method as described by the standard method of AOAC, 2005 [21].

### 2.7. Sample Extraction for the Determination of Antioxidant & Phytochemical Content

Prior to the analysis, samples were first extracted as described by [16], with slight modifications. 1.0 g of sample was mixed with 40 ml of 80% methanol in a mechanical shaker (model: Flash shaker—SF1 Stuart, S/S-R00102429) at room temperature for 12 hours. Extracts were filtered through Whatman filter paper (No. 1) and the filtrate was centrifuged (model: HERMLE Z306) at 3000 rpm for 10 minutes to obtain a clear supernatant. The stock solution (25 mg/ ml) was stored in amber coloured bottles at −3°C till further analysis.

### 2.8. Determination of Antioxidant Activity

#### 2.8.1. DPPH Radical Scavenging Activity

Radical scavenging activity of the extracts were measured using the methods described in [16, 24, 25], with modifications. 0.5 ml of various concentrations of *Moringa* extracts (12.5 mg/ml–0.78125 mg/ml) in methanol, were added to 2.0 ml of 30 mg/L solution of DPPH in methanol, having 0.980 (± 0.02) O.D. The mixtures were incubated for 30 min in the dark at room temperature and the absorbance was read at 517 nm, against a reagent blank. The control was made up of 0.5 ml methanol in 2.0 ml DPPH. The scavenging activity was estimated based on the percentage of DPPH radicals scavenged by using the following formula:

(3) $$\begin{array}{c} \%\text{Scavenging activity}=\left[\frac{\left(A_{\text{o}}-A_{\text{s}}\right)}{A_{\text{o}}}\right]\times 100, \end{array}$$

where, *A*_o_ = absorption of control, *A*_s_ = absorption of the test solution.

The concentration of the sample required for scavenging 50% of the DPPH free radical (EC_50_ value), was determined from the curve of percent scavenging plotted against the concentration. Each determination was done in triplicate and the average EC_50_ (mg/ml) value was obtained [26].

### 2.9. Determination of the Phytochemicals Content

#### 2.9.1. Total Phenolic Compounds Content

The total phenolic compounds content was determined using the Folin-Ciocalteu procedure as described by [26] with slight modifications. 0.2 ml of sample (25 mg/ml) was mixed with 0.5 ml of Folin-Ciocalteu reagent (diluted 1 : 10 with deionized water) and 2.5 ml of distilled water and it was allowed to stand for 3–8 min. Then 0.8 ml of Na_2_CO_3_ solution (7.5%, w/v) was added and the resultant solution was allowed to stand at room temperature for 30 min in a dark place. The absorbance was measured at 765 nm using the UV–Vis spectrophotometer. The total phenolic content was expressed as Gallic acid equivalents (GAE) in mg/100 g of the extract.

#### 2.9.2. Flavonoids Content

Total flavonoids were analyzed using the method described by [26] with slight modifications. 0.1 ml of sample (25 mg/ml) was added to 0.1 ml of 2% AlCl_3 _solution in methanol. The mixture was allowed to stand at room temperature for 10 min in a dark place. The absorbance of the mixture was measured at 415 nm against a blank sample without AlCl_3_ using the UV–Vis spectrophotometer. The flavonoid-Aluminium complex has an absorptivity maximum at 415 nm [27]. The total flavonoids content was expressed as Quercetin equivalents (QE) in mg/100 g of the extract.

### 2.10. Statistical Analysis

Data were presented as mean ± standard deviation (SD). All samples were run in triplicate *n* = 3. Values were statistically analyzed using one-way analysis of variance (ANOVA) and means were separated using Duncan's multiple range test using the SPSS.23 software package. A statistical probability (*p* value) less than 0.05 was regarded as significant. The mean ranking of the sensory scores were determined using the Friedman test.

## 3. Results & Discussion

### 3.1. Drying of Leaves

The total time taken by the *Moringa* leaves to completely dry were, 6 h at 55°C, 4.30 h at 60°C & 3 h at 65°C for unblanched leaves and 6 h at 55°C, 5.30 h at 60°C & 4 h at 65°C for steam blanched leaves (Table 1). These six samples were given random numbers as shown in parenthesis in Table 1. Out of the samples dried at the higher temperatures of 60°C and 65°C, steam blanched samples took one hour longer to dry than the unblanched samples. But both the samples (521 & 784); dried at 55°C, took the same time to dry. Steam blanching disrupts the cells and makes it easy for the moisture to evaporate. But since the steam blanched samples were cooled in cold water prior to drying, the initial moisture content of the disrupted cells were higher, thus increasing the time it takes to completely dry. If samples were not cooled in water prior to drying, the time it takes to dry would have been much less than in unblanched samples, since the moisture loss through disrupted cells is easy. In sample 784, since the time it takes to dry is longer compared to the other two steam blanched samples, the time & temperature would have been enough to completely dry this sample having disrupted cells with a higher initial moisture content, in comparison to the sample having intact cells with a lesser initial moisture content, thus equating the time those two samples took to completely dry.

### 3.2. Gross Yield Percentages, pH and Moisture Contents

There is a significant difference (*p* < 0.05) in gross yield between the samples. As shown in Table 2, the percentage yields of unblanched leaves (521, 592, 563) are higher than the steam blanched leaves (784, 725, 706). Steam blanching causes weight loss mostly due to softening of tissues [28]. Softening of tissues occur due to both turgor loss caused by cell membrane disruption and changes in cell wall polymers, for vegetables processed at high temperatures using steam (>90°C) [29]. Steam causes the cells to disrupt and water is evaporated from cells. This causes a large weight loss and also shrinkage can be seen lowering the masses and product yields. Carroad [30], has found that a 1.9% loss of product solids is seen for steam blanching and at the same time total losses of products cooled in water are higher than for the products cooled evaporatively without water.

pH is a parameter which depicts the sourness of the product [20]. Also pH of food materials gives an indication about the microbial safety of the food and a lower pH value indicates a higher microbial stability [31]. In this current research, there is a significant difference (*p* < 0.05) in pH, between the 6 samples shown in Table 2. As depicted in Table 2, the unblanched samples are slightly more acidic compared to the steam blanched samples. This slight acidity is due to the presence of phenolic, oxalic, gallic, and chlorogenic acids in *Moringa *[20, 26]. Soluble acids such as oxalic acids leach into water during cooling in water after the steam blanching treatment, thus causing a total increase in the pH of the product [31].

The moisture content of the fresh *Moringa oleifera *leaves were found to be 78.17 ± 1.57% (Table 3), which is slightly lower than 79.09 ± 1.00% and 80.04 ± 0.03% reported by Nobosse and Alakali [16, 32], but slightly higher than 72.83 ± 1.36% and 71.06 ± 0.07% reported by Rajput and Yameogo [33, 34]. Drying reduced the moisture content from 78% to less than 10%. Reducing the moisture content to a value less than 15% helps to prevent any microbial growth and improves the shelf life of the leaves because the microbial activity and autolysis is hampered at such low moisture contents. But it is considered satisfactory only for short term storage [14, 35]. According to research studies it has been found that a good quality tea should have a moisture content less than 7%. Tea with moisture in excess of 11% is liable to go mouldy. Thus moisture percentage should range from 6.1% to 9.2% for it to be satisfactory for long term storage [36]. In order to obtain a good quality herbal tea which is satisfactory for long term storage of about a year, the leaves were dried for less than 7% moisture content.

### 3.3. Colour Measurements

**Figure 1:**
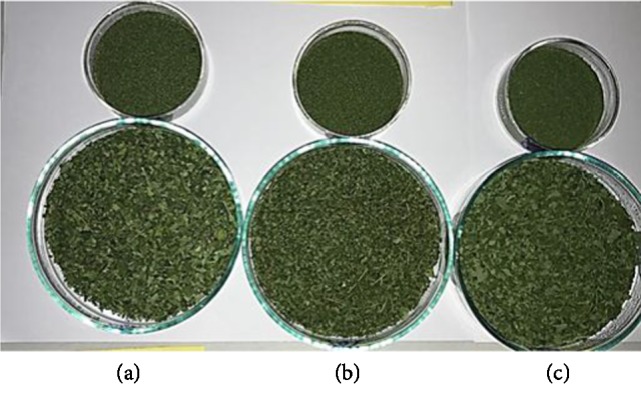
Unblanched *Moringa* leaves dried at (a) 55°C, (b) 60°C, (c) 65°C.

**Figure 2:**
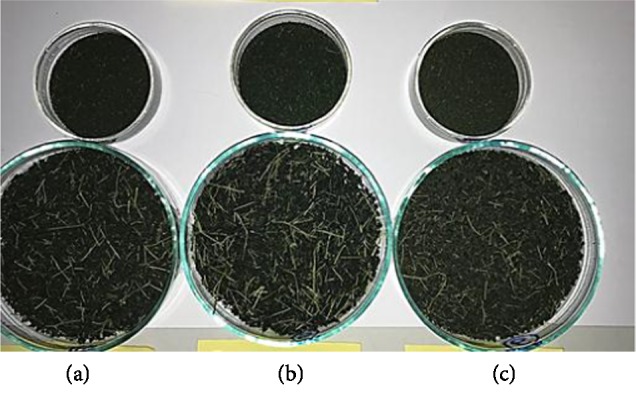
Steam blanched *Moringa* leaves dried at (a) 55°C, (b) 60°C, (c) 65°C.

The L^∗^ measures the lightness and darkness on a scale of 0–100, where “0” depicts blackness and “100” depicts whiteness. The a^∗^ values depicts greenness when negative and redness when positive and the b^∗^ values depicts blue when negative and yellow when positive [37]. The steam blanched samples are having lesser L^∗^ values (Table 4) compared to unblanched samples because the blanched samples are darker and intense green in colour as shown in Figure 2. Steam blanching causes the cells to disrupt and causes the air trapped in, to bubble away, so that it no longer clouds the colours, thereby giving a brighter green colour. Also when the steam blanched leaves are put in water to cool, the chloroplasts become swollen and may even burst and the green colour becomes more or less diffused though out the cell causing the more intense green colour on the surface of the leaves [38]. The blanched samples are having higher negative values for a^∗^ than for unblanched samples because the blanched samples are greener in colour than unblanched samples. Unblanched samples are having higher b^∗^ values indicating more yellowness in the samples. The natural green colour of leaves is due to a mixture of chlorophyll and during drying they are degraded to pyropheophytin and pheophytin [37]. Chlorophyll “a” appears blue-green in colour and chlorophyll “b” appears yellow-green in colour [39]. Chlorophyll “a” is less stable than chlorophyll “b”, so at higher temperatures chlorophyll “a” degrades more rapidly than chlorophyll “b”, giving rise to a dull yellow-green colour (Figure 1) as opposed to the natural green colour [37, 40].

### 3.4. Sensory Evaluation and the Quality Attributes

**Figure 3:**
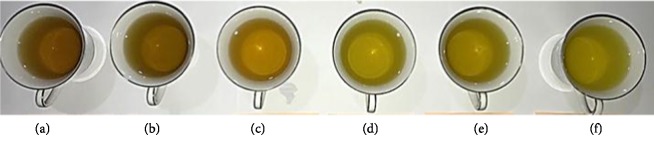
Brew colours of the****unblanched *Moringa* samples dried at (a) 55°C, (b) 60°C, (c) 65°C and steam blanched *Moringa* samples dried at (d) 55°C, (e) 60°C, (f) 65°C.

Table 5 illustrates the results of the sensory analysis conducted for the six *Moringa* samples. There is a significant difference (*p* < 0.05) between the samples for the dry leaf texture. Product 725, steam blanched sample, obtained the highest mean score for the dry leaf texture attribute. The consumer appetite for a certain food product is stimulated or dampened by its colour [13]. However the semi trained panelists scored in the range of average—like slightly, for the brew colours and there was no significant difference (*p* > 0.05) between the scores. But the highest score was given for the unblanched sample 592, followed by the steam blanched sample 725. As shown in Figure 3, the brew colours of the unblanched samples are somewhat brown in colour compared to the steam blanched samples, which are light yellowish in colour. This brown colour is due to the enzymatic browning reactions which occur in leaves, when phenolic compounds react with polyphenol oxidase (PPO) enzyme to produce complex polymeric compounds which are brown in colour. It is found that a short exposure to temperatures between 70 and 90°C is sufficient to inactivate the polyphenol oxidases [41]. Since the unblanched samples are only heated to less than 70–90°C, it is not sufficient to inactivate the PPO enzymes, thus gives the brown colour as shown in Figures 3(a)–3(c). However, Xiao [28] mentions that steam blanching effectively prevents the enzymatic browning reactions in vegetables. During steam blanching the leaves are heated to 100°C, which effectively inactivates the PPO enzymes, thus giving a pleasant light yellowish colour to the brew as shown in Figures 3(d)–3(f). Brew aroma has a great impact on the product acceptability [20]. However there was no significant difference (*p* > 0.05) between the samples for the brew aroma. But the steam blanched sample 706 obtained the highest mean score for brew aroma. Several studies have shown that taste is the major concerning factor in the acceptance and purchasing behavior of consumers [20]. The brew taste of the unblanched samples 521, 592 and 563 were significantly different (*p* < 0.05) from the steam blanched sample 706 and the highest mean score for the brew taste was observed in sample 706. According to Haslam [42], “astringency is generally recognized as a feeling of extreme dryness or puckeriness that is not confined to a particular region of the mouth or tongue, but is experienced invariably as a diffuse stimulus. Moreover, it may take a significant time to develop”. Steam blanched sample 784 was the most preferred in astringency. But there was no significant difference (*p* > 0.05) between the samples. Highest mean score for after taste was observed in the unblanched sample 563. However there was no significant difference (*p* > 0.05) between the mean scores. The overall acceptability of the unblanched samples 521, 592 and 563 were significantly different (*p* < 0.05) from the steam blanched sample 706 and the highest mean score for the overall acceptability was observed in sample 706.

According to the Friedman test ranks (Table 6), sample 706 (Steam blanched and dried at 65°C) gave the highest mean rank.

Also from the evaluation done by the trained panel of tea tasters, sample 706 was selected as the sample with the best sensory attributes. They analyzed that the brew of unblanched leaves was giving harsh unpleasant characteristics whereas the brew of the steam blanched leaves gave pleasant characteristics. Also they analyzed that the steam blanched dry leaf texture was blackish green and close to green tea dry leaf texture and was more favorable for a herbal tea.

Based on this outcome, proximate, mineral, vitamin, antioxidant and phytochemical analysis was done for the steam blanched sample dried at 65°C (sample 706) and the unblanched sample dried at 65°C (sample 563), in order to compare and contrast the effect of steam blanching on the nutritional quality.

### 3.5. Nutritional Composition

The proximate composition of unblanched and steam blanched *M. oleifera* leaves dried at 65°C is presented in Table 7. The results shows that all the parameters are significantly (*p* < 0.05) different from each other. The result shows that an acceptable level of moisture content is present in both samples. Such low moisture contents allow the storage of the samples at room temperature for an extended period of time. The values are in agreement with what was reported by [31], but lesser than the values reported by [6, 14, 42].

Ash % has significantly (*p* < 0.05) decreased in the steam blanched sample. Ash on food determines the extent of mineral matter likely to be found on the sample [23]. The decrease in the ash content with steam blanching could be due to leaching of minerals in to the cooling water, from the cells which are disrupted from steam [16]. Ash % of the steam blanched sample is in agreement with the reported values of [6, 23], and the ash % of the unblanched sample is in agreement with the reported value of [8].

Crude protein content of the steam blanched sample has significantly (*p* < 0.05) decreased compared to the unblanched sample. But the protein content of both these samples are very much higher than the protein content of alfalfa sprouts (3.7%), mung bean sprout (3.1%) and sweet potato leaves (3.3%) which are considered to be high protein vegetables [43]. Thus*, M. oleifera* leaves with a protein content of 32.26–39.07%, provide more than double the percentage provided by those vegetables. The protein content of the two samples are higher than the values reported by [6, 8, 14, 16, 22, 31, 32, 42]. *Moringa* is reported to have high quality protein which can easily be digested [22]. Fat in food is a determining factor for the amount of energy available [23].

The crude fat content has significantly (*p* < 0.05) increased in the steam blanched sample. The fat content of these two samples are higher than the reported values by [6, 8, 14, 31], but are in agreement with the values reported by [22, 42]. Steam blanching significantly (*p* < 0.05) reduced the fiber content. The values of the two samples were lesser than the values reported by [6, 8, 14, 22, 31]. The carbohydrate and calorie content of the steam blanched sample was significantly (*p* > 0.05) higher than the unblanched sample, making the steam blanched sample a better source of energy. Alakali [31], has found dry leaves to be a better source of protein, fat, fiber and carbohydrate than the fresh leaves.

### 3.6. Mineral Composition

Data represented in Table 8 illustrates the mineral content of the two *Moringa* samples 563 & 706. The minerals Mg, Zn and Cu didn't show any significant difference (*p* > 0.05) between the two samples whereas the minerals Na, K, Ca, Mn, and Fe showed significant difference (*p* < 0.05) between the two samples. Na, K and Ca, shows a significant decrease in the steam blanched sample whereas Mn and Fe have significantly increased in the steam blanched sample. The Ca content has been found in very high concentration, followed by K and Mg in both the samples and this is in agreement with Yameogo [33]. Certain mineral contents in this study were in the same range as reported in certain studies, whereas certain mineral contents had variations [6, 8, 14, 22, 23, 31, 33]. According to [6, 8, 44], these variations in the mineral contents of the *Moringa* leaves from different regions is due to the availability of soil nutrients, application of fertilizers to the soil, cultivated regions, growing conditions, seasonal changes and the nature of soil. Olabode [14] found that the mineral content reduced significantly with the increase in temperature, but Alakali [31] has shown that an increase in drying temperature do not deplete the mineral content. The decrease in the minerals Na, K and Ca of the steam blanched sample could be due to the leaching of minerals in to the cooling water, from the cells which are disrupted from condensed steam [16]. Na & K are helpful in the transmission of nerve impulses and maintaining the electrolyte balance [8]. Ca is required for teeth and bone development, and also in the transport of oxygen, whereas a deficiency in this could cause rickets, bone pain and muscle weakness [8, 23, 31]. Mg is involved in functioning of about 90 enzymes in the body [8]. Mn is required for the building of the immune system and regulation of blood sugar levels [23]. Cu is required for the enzyme production and Zn plays a vital role in gene expression, protein and nucleic acids metabolism, and acts as a co-enzyme for carbohydrates [23].

### 3.7. Vitamin Content

Table 9 shows the Vitamin C, Vitamin A and Vitamin E contents of the unblanched and steam blanched *Moringa *samples dried at 65°C. There is no significant difference (*p* > 0.05) between the two samples in the vitamin C content whereas there is a significant difference (*p* < 0.05) between the two samples in the vitamin A and vitamin E contents.

Even though there is no significant difference in the vitamin C content between the two samples, there is a slight decrease in the vitamin C content of the steam blanched sample. This is due to the fact that vitamin C, not being stable at high temperatures and it being very soluble in water. Thus the high temperature during steam blanching could have inactivated some of the vitamin C, and water used for cooling after steam blanching could have washed away some vitamin C as well [15, 16]. Clement [17], states that *Moringa oleifera* leaves should be dried at a temperature lower than 70°C, to avoid the damage caused by the heat. Thus the temperature of 65°C is in the safe range to retain the vitamin C content effectively. Vitamin C is involved in the synthesis of connective tissues, helps to increase the iron absorption in the body, defends the human body from oxidative stress and is very important as an antioxidant [19, 20, 44].

Vitamin A content has significantly increased in the steam blanched sample. This is in agreement with the findings of [15, 16]. This increase in blanched sample is due to the breakdown of the cellulose structure and the thermal disruption of the noncovalent associations which allows the extraction of vitamin A more effectively and also due to vitamin A being a fat soluble vitamin [16, 17]. In plant materials, vitamin A is available as provitamin A. They convert beta-carotene: the most potent precursor of vitamin A, to provitamin A within the body [16, 41]. Beta-carotene is the main source of vitamin A in fruits & vegetables [45]. Dutra-de-olivera [46] found that, there was no loss of carotene at 100°C. Thus blanching doesn't destroy beta-carotene, but rather increase the total solid matter content due to the lower moisture content than unblanched leaves [15]. Ihns [47] found that beta-carotene content increased with the increase in temperature because the solubility increased at high temperature, but a longer drying time reduced the beta-carotene content significantly because of the oxidation with air. Vitamin A is a powerful antioxidant. It helps in maintaining healthy vision, immune system, neurological function, healthy skin and also prevents aging and cancer [15, 17]. Deficiency in vitamin A causes blindness, which ranges from impaired dark adaptations to night blindness. *Moringa* leaves can help delay development of cataract, prevent night blindness and also eye problems in children [10]. Thus this *Moringa *herbal tea could be a valuable source in overcoming the problem of vitamin A deficiency.

Vitamin E content is significantly higher in the steam blanched sample, due to the higher extractability from the disrupted and broken down cellulose structure. Vitamin E has well established antioxidant properties and helps the body to destroy free radicals [44]. It also assists animals to develop disease resistance and to maintain and increase the storage of vitamin A and iron in the body [41].

### 3.8. Antioxidant Potential

The antioxidant potential of the *Moringa oleifera* samples were measured using the DPPH radical scavenging assay. There was a gradual increase in DPPH absorbance and it implies a decreasing antioxidant potential with decreasing concentration of the sample [48]. Thus the results in Table 10 reveals that sample 563 and 706 have radical scavenging properties with different degrees of potentials. DPPH has a hydrogen free radical and the *Moringa* samples are able to reduce the unstable radical DPPH to diphenylpicrylhydrazine through proton donation [26, 42]. Phenolic compounds are the major compounds responsible for the antioxidant activity in plant materials and it has been found that higher the total polyphenols, higher is the antioxidant activity [9, 42]. This could be one reason, for the steam blanched sample to be having a higher DPPH radical scavenging activity, than the unblanched sample as shown in Table 10. That is because during steam blanching the polyphenol oxidase enzymes are inactivated, thus increasing the phenolic content, which would consequently increase the antioxidant potential. This increase could also be explained by the fact that steam blanching enhances the extractability through matrix softening [49].

In regard to the EC_50_ value, the unblanched sample and the steam blanched sample showed significantly (*p* < 0.05) different values of 3.48 and 2.08 mg/ml, respectively. EC_50 _value is the concentration of the sample, required to scavenge 50% of the DPPH free radicals [26]. Thus it is obvious from the results that a lower concentration of sample is adequate to scavenge the DPPH radicals using the steam blanched samples whereas in the unblanched sample it requires a somewhat higher concentration.

Atawodi [50], found that all parts of the *Moringa* plant, displays very strong antioxidant/radical scavenging activity, but the leaves has the highest radical scavenging potential. Thus, this *Moringa* herbal tea made out of leaves could be very beneficial in prevention of oxidative stress, cardiovascular diseases, cancer and onset of diseases [8, 42, 48].

### 3.9. Phytochemical Content

Phenolic compounds are phytochemicals in plant tissues of fruits and vegetables. They are secondary metabolites synthesized through the shikimic and phenylpropanoid pathways and possess numerous bioactive properties. The intake of them provides numerous health-protective effects, even though phenolic compounds are not considered as nutrients [51]. There are more than 8000 individual plant phenolic compounds and they are divided in top two main groups as flavonoids and nonflavonoids. The nonflavonoids contain compounds like stilbens, lignans, volatile phenols, phenolic acids, quinones, etc. [51]. Total phenolic content (TPC) and the flavonoids content (FC) of the two samples, 563 and 706 are show in Table 11. These values are higher than the TPC contents reported by [42, 52], but are somewhat lower than the values reported by [8, 16]. The TPC in the unblanched sample is significantly (*p* < 0.05) higher than the steam blanched sample. This reduction of the phenolic content could be due to the leaching of phenolic compounds in to water during cooling, from the disrupted cells. Phenolic compounds contribute to the colour, flavor and astringency of vegetables [20]. Phenolic compounds are known to possess antioxidant potential, hypoglycemic, hypolipidemic, anti-tumor and antidiabetic properties [52]. Also phenolic compounds are believed to be the major group responsible for antioxidant activity and have an important role in stabilizing lipid oxidation [42].

The flavonoids content (Table 11) of the steam blanched sample is somewhat lower than in the unblanched sample. FC content in these two samples seems to be very much lower compared to the TPC content. FC is lower than the values reported by [8, 52]. Flavonoids have long been recognized to possess anti-inflammatory, anti-allergic, antiviral, anti-proliferative and anti-carcinogenic activities [53]. In the study done by Nweze [53], it was found that the aqueous extract constituted more phytochemicals than the ethanolic extract, thus rendering water a good solvent for the extraction of phytochemicals from *Moringa *leaves. Also, Komes [1] found that a maximum extraction efficiency of bioactive compounds from green tea is achieved during aqueous extraction at 80°C, for five minutes. Thus, it is evident that brewing the *Moringa* herbal tea for five minutes using water heated to 80°C, would give the maximum health benefits.

## 4. Conclusion

This study shows the *Moringa* herbal tea product 706, to be the most preferred in terms of brew aroma, brew taste and overall acceptability. Based on the results the best fit time-temperature combination for drying was found to be 65°C for 4 hours and the most suitable sample preparation method was found to be steam blanching.

Steam blanching significantly reduced the crude protein and crude fiber contents but significantly increased the fat and carbohydrates content. Steam blanching significantly decreased the Na, K and Ca content whereas it increased the Mn & Fe contents. But Zn, Cu & Mg were unaffected by steam blanching. Ca was found in very high concentration, followed by K & Mg in both the samples 563 & 706. This study suggests *Moringa* herbal tea to be a very good source of nutrients & minerals.

Steam blanching did not significantly affect the vitamin C content and the temperature of 65°C was in the safe range to retain the vitamin C content effectively. Vitamin A and Vitamin E contents were significantly increased by steam blanching. Antioxidant activity was higher in the steam blanched sample. Total phenolic content and the flavonoids content were somewhat reduced by steam blanching. But the extraction efficiency could be increased by brewing the herbal tea at 80°C for five minutes. The *Moringa* herbal tea produced by steam blanching and drying at 65°C for 4 hours could thus be beneficial as a functional food with numerous health benefits and therapeutic effects.

## Figures and Tables

**Table 1:** Time, temperature combinations.

Temperature (°C)	Time (h)	
	Unblanched	Steam blanched
55	6 (521)	6 (784)
60	4.30 (592)	5.30 (725)
65	3 (563)	4 (706)

*n* = 3.

**Table 2:** Gross yield percentages & pH.

Sample	Unblanched			Steam blanched		
	521	592	563	784	725	706
Gross yield (%)	23.33 ± 0.34	22.64 ± 0.34	20.91 ± 0.54	14.72 ± 0.45	19.31 ± 0.21	15.62 ±0.71
pH	5.95 ± 0.01	5.91 ± 0.01	5.93 ± 0.00	6.33 ± 0.01	6.24 ± 0.01	6.27 ± 0.01

*n* = 3; mean ± SD.

**Table 3:** Moisture percentages (dry basis).

Sample	Fresh Leaves	Unblanched			Steam blanched		
		521	592	563	784	725	706
Moisture %	78.17 ± 1.57 (dry basis)	6.64 ± 0.25	5.63 ± 0.19	4.29 ± 0.10	5.35 ± 0.06	7.03 ± 0.02	5.48 ± 0.42

*n* = 3; mean ± SD.

**Table 4:** The L^∗^, a^∗^ and b^∗^ values of the dried leaves and powder.

Sample	L^∗^		a^∗^		b^∗^	
	Leaves	Powder	Leaves	Powder	Leaves	Powder
521	21.09 ± 3.33	24.06 ± 1.63	−4.39 ± 0.87	− 4.26 ± 2.63	12.93 ± 1.20	15.28 ± 0.71
592	20.84 ± 2.94	20.29 ± 1.86	−3.91 ± 0.70	−4.16 ± 0.35	12.51 ± 1.27	13.59 ± 0.75
563	22.59 ± 3.52	21.04 ± 1.98	−3.99 ± 0.71	−4.01 ± 0.32	11.56 ± 1.64	14.13 ± 0.95
784	8.39 ± 4.97	9.14 ± 6.50	−1.53 ± 0.31	−1.97 ± 0.40	4.59 ± 2.42	3.35 ± 3.17
725	12.71 ± 2.03	13.96 ± 0.90	−1.84 ± 0.60	−2.23 ± 0.24	5.05 ± 1.14	6.13 ± 0.47
706	7.31 ± 4.70	6.94 ± 4.80	−1.16 ± 4.09	−1.68 ± 0.48	2.52 ± 2.62	3.34 ± 2.32

*n* = 3; mean ± SD.

**Table 5:** Quality attributes of the sensory evaluation.

Quality attribute	Unblanched			Steam blanched		
	521	592	563	784	725	706
Dry leaf texture	2.57^a^ ± 0.85	2.49^a^ ± 0.98	3.06^b^ ± 1.06	3.74^c^ ± 0.98	**3.97^c^** **±** **1.01**	3.69^c^ ± 0.96
Brew colour	3.14^a^ ± 1.14	**3.37^a^** **±** **1.11**	3.23^a^ ± 1.11	3.23^a^ ± 1.26	3.34^a^ ± 0.87	3.31^a^ ± 1.16
Brew aroma	2.77^a^ ± 1.14	3.00^a^ ± 1.11	3.20^a^ ± 1.10	3.14^a^ ± 1.22	3.09^a^ ± 1.31	**3.26^a^** **±** **1.38**	
Brew taste	2.57^a^ ± 1.27	2.63^a^ ± 1.06	2.80^a^ ± 1.27	3.09^ab^ ± 1.31	3.17^ab^ ± 1.27	**3.49^b^** **±** **1.27**
Astringency	2.80^a^ ± 1.35	2.77^a^ ± 1.24	2.91^a^ ± 1.44	**3.09^a^** **±** **1.31**	2.83^a^ ± 1.04	2.97^a^ ± 1.12
After taste	2.40^a^ ± 1.29	2.71^a^ ± 1.23	**2.91^a^** **±** **1.48**	2.77^a^ ± 1.19	2.83^a^ ± 1.01	2.83^a^ ± 1.20
Overall acceptability	2.71^a^ ± 1.23	2.69^a^ ± 1.02	2.97^ab^ ± 1.15	3.17^ab^ ± 1.40	3.23^ab^ ± 1.14	**3.51^b^** **±** **1.40**

*n* = 35; mean ± SD. Values in a row followed by different letters in superscript are significantly different (*p* < 0.05). The values in bold represent the highest mean score for each attributes. And the significance was compared for the six samples for each attribute, i.e., row wise. For example, for dry leaf texture the letters “a”, “b”, “c” represents the significant difference among the six samples. The superscript “ab” in brew taste represents an intermediate condition.

**Table 6:** Friedman test ranks.

Sample	521	592	563	784	725	706
Mean rank	2.97	3.00	3.53	3.53	3.97	**4.19**

*n* = 35. The mean ranking of the sensory scores were determined using the Friedman test. The value in bold represents the sample with the highest mean rank. Friedman test is a ranking system to find the sample with the best sensory attributes. So according to the results sample 706 portrays the best rank. Since this is a ranking system, significance is not calculated.

**Table 7:** Proximate composition of unblanched and steam blanched *M. oleifera* leaves dried at 65°C.

Proximate composition (db)	Unblanched sample (563)	Steam blanched sample (706)
Moisture (%)	4.29^a^ ± 0.10	5.48^b^ ± 0.42
Ash (%)	10.66^b^ ± 0.02	6.74^a^ ± 0.04
Crude protein (%)	39.07^b^ ± 0.68	32.26^a^ ± 1.36
Crude fat (%)	6.58^a^ ± 0.27	7.83^b^ ± 0.65
Crude fiber (%)	8.03^b^ ± 0.03	7.05^a^ ± 0.02
Carbohydrates (%)	31.37^a^ ± 0.97	40.64^b^ ± 1.63
Calorie (%)	341.00^a^ ± 1.39	362.07^b^ ± 4.68

*n* = 3; mean ± SD. db = dry basis. Values in a row followed by different letters in superscript are significantly different (*p* < 0.05).

**Table 8:** Mineral concentrations of unblanched and steam blanched *M. oleifera* leaves dried at 65°C.

Mineral composition (mg /100 g)	Unblanched sample (563)	Steam blanched sample (706)
Na	31.99^b^ ± 0.88	15.24^a^ ± 4.22
K	858.52^b^ ± 110.11	445.22^a^ ± 61.40
Ca	871.48^b^ ± 73.47	627.01^a^ ± 34.73
Mg	244.94^a^ ± 30.51	265.38^a^ ± 43.91
Mn	51.11^a^ ± 3.36	132.29^b^ ± 20.29
Zn	1.71^a^ ± 0.34	1.20^a^ ± 0.06
Fe	19.76^a^ ± 1.55	64.38^b^ ± 21.67
Cu	1.63^a^ ± 1.48	0.89^a^ ± 0.57

*n* = 3; mean ± SD. Values in a row followed by different letters in superscript are significantly different (*p* < 0.05).

**Table 9:** Mineral concentrations of unblanched and steam blanched *M. oleifera* leaves dried at 65°C.

Vitamin	Unblanched sample (563)	Steam blanched sample (706)
Vitamin C (mg/100 ml)	49.00^a^ ± 23.13	44.80^a^ ± 2.42
Vitamin A (mg/100 g)	3.37^a^ ± 0.01	3.68^b^ ± 0.11
Vitamin E (mg/100 g)	86.87^a^ ± 0.06	93.77^b^ ± 0.06

*n* = 3; mean ± SD. Values in a row followed by different letters in superscript are significantly different (*p* < 0.05).

**Table 10:** Scavenging effect of DPPH radicals using unblanched and steam blanched *M. oleifera* leaves dried at 65°C.

Antioxidants	Unblanched sample (563)	Steam blanched sample (706)
DPPH scavenging %	55.98^a^ ± 0.92	62.96^b^ ± 1.19
EC_50_ (mg/ml)	3.48^b^ ± 0.23	2.08^a^ ± 0.39

*n* = 3; Mean ± SD. Values in a row followed by different letters in superscript are significantly different (*p* < 0.05).

**Table 11:** Phytochemical contents of unblanched and steam blanched *Moringa* leaves dried at 65°C.

Phytochemical content	Unblanched sample (563)	Steam blanched sample (706)
Total phenolic content (mg GAE/100 g)	2042.9^b^ ± 16.7	1916.9^a^ ± 17.2
Flavonoids content (mg QE/100 g)	21.21^b^ ± 0.24	19.07^a^ ± 0.70

*n* = 3; mean ± SD. Values in a row followed by different letters in superscript are significantly different (*p* < 0.05), GAE: gallic acid equivalents, QE: quercetin equivalents.

## Data Availability

The data used to support the findings of this study are included within the article.
